# Detection of Lip, Tongue, Masseter, and Temporalis Muscle Contractions with Electromyography Tools as an Early Diagnostic Medium for Dentoalveolar Malocclusion

**DOI:** 10.1055/s-0044-1795124

**Published:** 2024-12-30

**Authors:** Harun Achmad, Intan Sari Areni, Sumintarti Sumintarti, Sri Ramadhany, Reza Ardiansya, Sarwo Edy, Wesley Kuandinata

**Affiliations:** 1 Department of Pediatric Dentistry, Faculty of Dentistry, Hasanuddin University, Makassar, Indonesia; 2 Departement of Electrical Engineering, Faculty of Engineering, Hasanuddin University, Makassar, Indonesia; 3 Department of Oral Medicine, Faculty of Dentistry, Hasanuddin University, Makassar, Indonesia; 4 Department of Public Health and Preventive Medicine, Faculty of Medicine, Hasanuddin University, Makassar, Indonesia

**Keywords:** malocclusion, muscle contractions, electromyography

## Abstract

**Objectives:**

This study aims to detect early class I, II, and III malocclusions through the muscle strength of the lips, tongue, masseter, and temporalis.

**Materials and Methods:**

The study subjects were 30 pediatric patients with predetermined criteria. The subjects were divided into class I, II, and III malocclusions where each classification of malocclusion amounted to 10 people. Subjects were differentiated according to gender and age. Tongue pressure during swallowing was recorded by a palatal measurement sensor system. The strength of the activity was assessed when the lip muscles resisted as hard as possible the traction plate placed between the teeth and the lips, then the force was connected to an electromyograph (EMG) to be measured. Temporal and masseter muscle contractions were assessed when the muscles performed swallowing, chewing, mouth opening, resting, mouth closing, and biting movements.

**Statistical Analysis:**

Data analysis using the SPSS application was performed with the ANOVA test if the data distribution was normal, and if the data distribution was not normal, then the Kruskal–Wallis test was used. Significant data were evaluated by post-hoc tests using least significant difference if the data distribution was normal or the Mann–Whitney test if the data distribution was not normal.

**Results:**

It was found that there was a significant difference in the left masseter muscle and left temporalis muscle.

**Conclusion:**

EMG can be considered as a tool to detect class I, II, and III malocclusions through muscle contraction. Biting and chewing positions have satisfactory EMG examination results for malocclusion detection. Age and gender of the child may affect the results of EMG examination in certain conditions.

## Introduction

The stomatognathic system is the system that makes up the oral cavity. The components of the stomatognathic system consist of the maxillary and mandibular bones, temporomandibular joints and ligaments, masticatory muscles, and periodontal structures that work together to perform chewing, swallowing, and phonatory functions. 1 Chewing, swallowing, and phonatory functions involve the orofacial muscles which consist of the masticatory muscles, cheek and lip muscles, soft palate muscles, suprahyoid muscles, and tongue muscles. 2

Biomechanical or functional disorders of the stomatognathic system such as malocclusion, temporomandibular disorders, chewing, and swallowing abnormalities can have a direct impact on the masticatory muscles causing muscle asymmetry and altering head posture through muscle sequences and adaptation mechanisms in posture. 3

Malocclusion can be caused by the absence of dentofacial balance. Imbalance is influenced by several factors: genetic, environmental, growth and development, ethnic, functional, and pathology. 4 The etiology of malocclusion from environmental factors after tooth eruption can be influenced by muscle strength activity, muscle position at rest, sucking habits, and abnormalities in mandibular movement from resting position to centric position, which in every mandibular activity involves the muscles of mastication. 5 6

Assessment and measurement of muscle activity can be obtained from electromyography (EMG), which is an experimental technique for assessing muscle activity. 7 Studies on lip muscle activity with the use of EMG found significant differences in conditions of dentofacial imbalance in the swallowing state. 8 In measuring the strength of the tongue muscles, a device that has a sensor in the palatal region during swallowing is used. 9

EMG is a technique for measuring, recording, and analyzing myoelectric signals from muscle activity. 10 11 12 13 14 15 This technique can be used to assess the activity of facial and masticatory muscles. An understanding of the neuromuscular anatomy and physiology of the muscles to be explored is necessary for the analysis and interpretation of EMG in dental practice as a method of static and dynamic functional investigation of the motor units included in the orofacial muscles in general and those that move the mandible, especially the masseter muscles, temporalis muscles, orbicular lip, buccal, tongue, and suprahyoid muscles involved in mastication and swallowing. 15 16 17 18 19 20 These muscles not only affect facial shape but also impact malocclusion and tooth location. 21 22 23 24

The structural core of EMG is the motor unit. The total extracellular potential of the motor unit muscle fiber action potential is known as the motor unit action potential. The muscle motor unit is the most fundamental component. The motor unit consists of a motor neuron and each muscle fiber that can be innervated by an axonal branch of the motor neuron. The electrical signal generated when the muscle fibers of the motor unit are triggered, known as the motor unit action potential, is monitored by electrodes. 18 25 EMG has an important role in evaluating the relationship between malocclusion and muscle activity; it is the determination of the electrical activity of muscle tissue or its depiction as a visual display or signal by using electrodes connected to the skin or implanted into the muscle. 26 27 28

Research based on EMG tools to detect muscle contractions has been performed by several researchers, but only to see changes in muscle contractions after treatment and not to detect malocclusion. 6 10 20 29 30 Based on several supporting studies, there is a possibility of differences in contraction of the lip, tongue, masseter, and temporalis muscles in children with class I, II, and III malocclusion.

## Materials and Methods

The type of research used was analytic observational with a cross-sectional study design. The research location was Department of Pediatric Dentistry at Hasanuddin University Dental and Oral Hospital.

This study was prospective and was approved by the Health Research Ethics Committee of the Dental and Oral Hospital FKG UNHAS (0122/PL.09/KEPK FKG-RSGM UNHAS/2023). Consent from the participants was obtained by filling in the consent form. This study began in June to August 2023. In this study, the total sample was 30 children who were divided into three groups (class I malocclusion group, class II malocclusion group, and class III malocclusion group).

Data analysis using the SPSS application was performed with the ANOVA test if the data distribution was normal, and if the data distribution was not normal then the Kruskal–Wallis test was used. Significant data were evaluated by post-hoc tests using least significant difference if the data distribution was normal or the Mann–Whitney test if the data distribution was not normal.

**Fig. 1:**
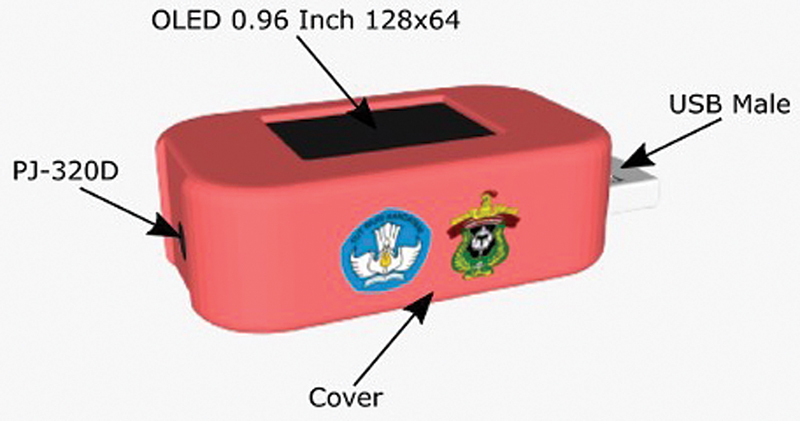
Dentosmart electromyography.

**Fig. 2:**
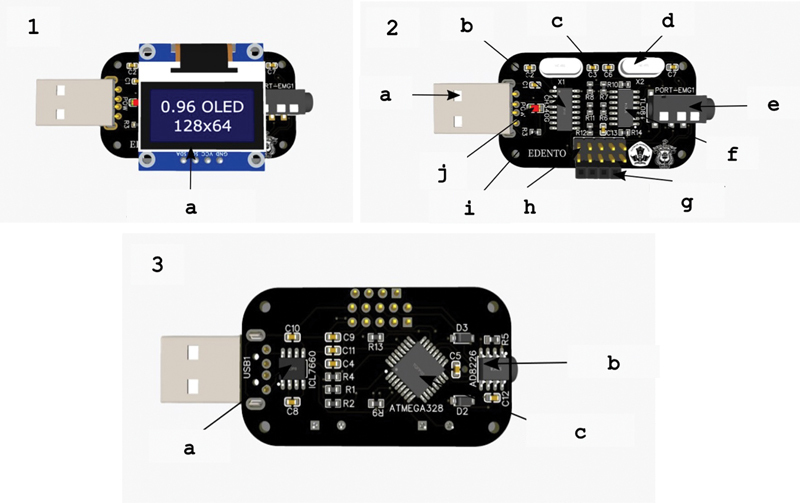
(1) PCB design layout E-Dento top view with its main component, a 0.96 Inch 128 × 64 OLED. (2) PCB design layout E-Dento top view without OLED and its main components are: (a) USB male; (b) CH340G; (c) capacitor: Menstabilkan tegangan dan memfilter frekuensi dari electrode yang telah dikuatkan; (d) Xtall; (e) PJ-320D; (f) Stabilizes voltage and filters frequency from electrode; (g) TL084IDR; (h) female header; (i) male header; (j) resistor; (k) LED. (3) PCB design layout E-Dento bottom view: (a) ICL7660M; (b) AD8226BRMZ-R7; (c) ATMEGA328P-AU.

**Fig. 3:**
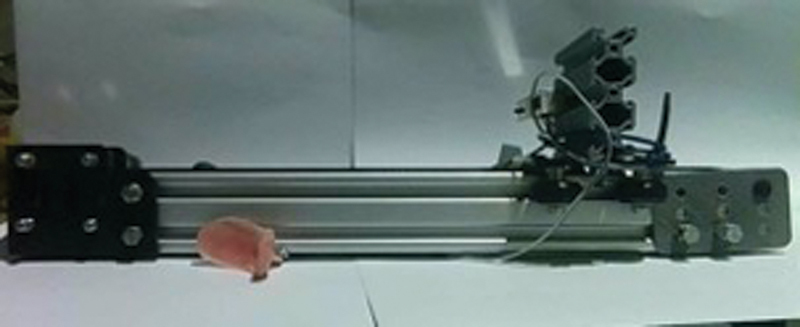
Tongue smart for measuring muscle contraction pressure.

**Fig. 4:**
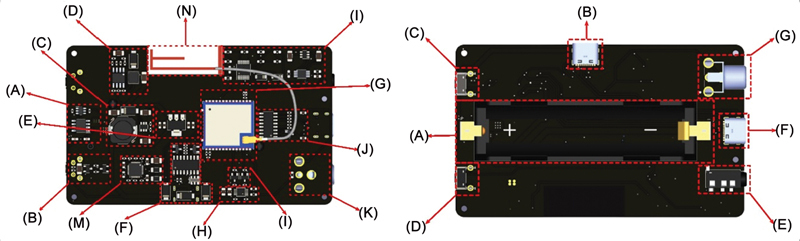
PCB tongue smart top and bottom views. Description: (A) full system schematic circuit; (B) battery management system; (C) power latch system; (D) boost converter; (E) inverting buck boost converter; (F) +3V3 voltage regulator; (G) power charging input and USB to TTL; (H) microcontroller unit (MCU); (I) external ADC; (J) I2C level shifter; (K) sensor.

Inclusion criteria are pediatric dentistry patients aged 6 to 17 years who come to the dental hospital, children who have never received orthodontic treatment, permanent first molar teeth have erupted, and dentoalveolar malocclusion criteria. Exclusion criteria were craniofacial abnormalities, congenital abnormalities, and children with special needs. Patients' malocclusion was diagnosed according to Angle's classification looking at the relation of the first permanent molar. The Dentosmart device was utilized in this study to measure the contraction of the masseter and temporalis muscles during resting, biting, chewing, swallowing, and opening and closing their mouth ( Figs. 1 and 2 ). A tongue smart device was used to record, read, and measure the strength of lip and tongue muscle contractions. ( Figs. 3 and 4 ).

### Surface Electrodes

The working mechanism of the Dentosmart EMG device consists of several components: surface electrodes as signal receivers of masseter, temporalis, and mentalis muscles; battery as power supply; Raspberry as a single board circuit; Android smartphone as a display of diagnostic results using the application; EMG as a muscle electrical activity recorder and IC (integrated circuit) MCP3008 as a signal converter; a 6.5-inch LCD as an interface display of the data-operating system. Using a silver/silver chloride (Ag/AgCl) adhesive with bipolar electrode surfaces (Noraxon Dual, Scottsdale, Arizona, United States), the EMG device circuit was applied to the skin in the chin region (mental muscles), on the left and right superficial masseter muscles parallel to the muscle fibers, and on the left and right anterior temporal muscles.

The invention used is tongue smart, which is a device with three sensors, each sensor has a different measuring function, so there are three different sensor ports on the device. The sensors used in tongue smart consist of: (1) a force sensing resistor to measure the pressure generated by the tongue with kPa measuring unit; (2) a load cell to measure the pressure that can be held by the lips with kPa measuring unit; (3) EMG to measure the strength of facial muscle contractions with micro-Volt measuring units. The measurement results obtained by each sensor will be sent to the microcontroller unit to be processed and then sent to the monitoring place (laptop or computer) to display data from each sensor.

Tongue pressure during swallowing is recorded by a sensor sheet system with five measurement points attached to the palatal mucosa. The time sequence, maximum magnitude and duration of tongue pressure, and swallowing time were analyzed. The recorded tongue pressure waveforms were used to evaluate the tongue for each measurement point and swallowing time. 9

### Patient Instructions

Instructions are given verbally to the patient before recording surface EMG readings. The head and body should not be moved during recording of EMG values, as even slight movements can affect the recording output. The tongue should be kept still and in position, as its movement will result in stimulation of other oral muscles which will alter the recording results. Before inserting the electrodes, the skin area intended for the EMG was cleaned with 70% ethyl alcohol and dried with cotton swabs.

### EMG Recording and Reading Procedure

The masseter and temporalis muscles can be recorded and read by the operator using the Dentosmart device's surface EMG. The muscle surface was covered in gel electrodes. Three surface leads on each side are used to measure muscle activity. Of these, one served as a reference electrode and the other two as recording electrodes. The electrodes are placed over the muscle to be recorded based on the position of the contraction, which is felt. It is emphasized to the patient to adhere to all guidelines. EMG measures the contraction of the masseter and temporalis muscles when the masseter muscle is used for swallowing, chewing, opening the mouth, or resting.

The patient was instructed by the lip muscle assessment to hold the plate between their lips and teeth, which the tongue smart device would retract automatically. The patient was instructed to place their tongue on the pressure-sensitized palate area by the tongue muscle assessment.

## Results

**Fig. 5:**
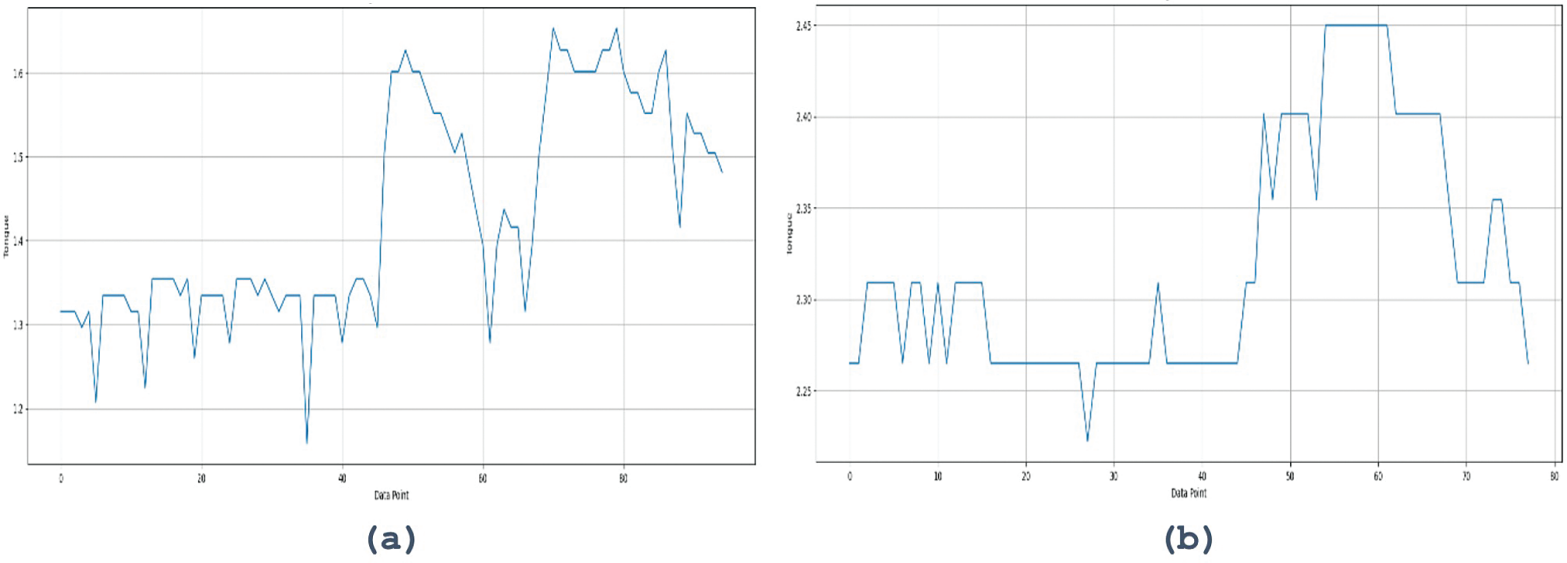
EMG display of tongue muscle strength on tongue smart device. EMG, electromyography.

The characteristics of the respondents provide an overview of the respondents who are the subjects of the study. The number of male and female samples was 10 and 20 samples. The distribution of research samples was as follows: age 6 to 12 years amounted to 18 samples and age >12 years amounted to 12 samples. Tables 1 and 2 present the findings from the measurement and data analysis of lip and tongue muscle contractions based on gender and malocclusion as investigated. Table 3 displays the results of the measurement based on age. Figs. 4 and 5 show the EMG results of tongue and lip muscle contractions using the tongue smart.

**Table 1:** Data analysis of lip muscle and tongue muscle contraction data by malocclusion group

Muscle contraction	Malocclusion	Mean (Pa)	Standard deviation	*p* -Value
Lip	Class I	29.07	13.47	0.491 a
	Class II	36.09	18.54	
	Class III	35.67	15.53	
Tongue (Point 1)	Class I	8.81	14.51	0.328 a
	Class II	5.49	8.12	
	Class III	6.55	8.58	
Tongue (Point 2)	Class I	6.36	12.68	0.760 a
	Class II	6.57	12.55	
	Class III	5.76	8.93	
Tongue (Point 3)	Class I	2.54	2.30	0.612 a
	Class II	5.65	8.31	
	Class III	2.89	1.63	
Tongue (Point 4)	Class I	6.34	6.28	0.968 a
	Class II	5.25	5.01	
	Class III	6.24	6.27	

Abbreviation: Pa, Pascal.

a Data analysis showed *p* >0.05, which means it is not significant.

**Table 2:** Data analysis of the strength of contraction of the tongue and lip muscles based on gender and age

Muscle	Male		Female		*p* -Value	6–12 years		> 12 years		*p* -Value
	Mean (Pa)	SD	Mean (Pa)	SD		Mean (Pa)	SD	Mean (Pa)	SD	
Tongue 1	7.74	9.45	6.64	11.15	0.387 a	7.18	8.68	6.63	13.12	0.451 a
Tongue 2	11.18	15.42	4.33	8.73	0.266 a	6.40	11.31	6.00	11.41	0.797 a
Tongue 3	5.08	7.18	3.16	4.14	0.572 a	4.92	6.36	1.98	1.55	0.070 a
Tongue 4	6.59	5.02	5.70	6.06	0.237 a	5.41	5.63	6.69	5.99	0.480 a
Lip muscle	26.89	21.19	36.19	12.87	0.219 a	32.16	16.05	35.64	15.92	0.525 a

Abbreviations: Pa, Pascal; SD, standard deviation.

a Data analysis shows *p* >0.05, which means it is not significant.

**Table 3:** Data analysis of temporalis and masseter muscles on various movements

Muscles	Rest	Closing the mouth	Biting	Chewing	Swallowing	Opening the mouth
**Right temporal**	0.198	0.517	0.723	0.448	0.491	0.056
**Left temporal**	0.029 a	0.017 a	0.041 a	0.036 a	0.140	0.149
**Right masseter**	0.617	0.331	0.384	0.251	0.131	0.053
**Left masseter**	0.025 a	0.390	0.009 a	0.039 a	0.027 a	0.153

a Data analysis shows *p* <0.05, which means it is significant *.*

**Fig. 6:**
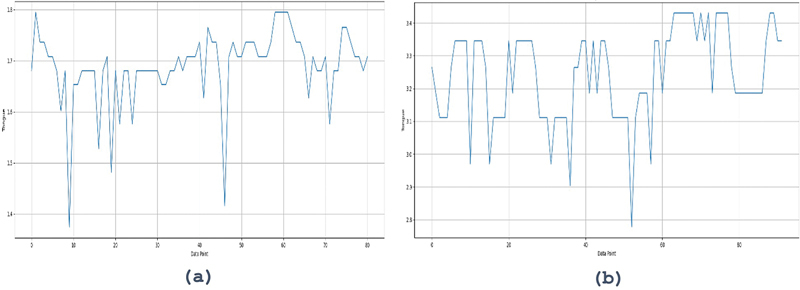
EMG display of lip muscle strength on tongue smart device. EMG, electromyography.

Table 1 shows that there is no significant difference in lip and tongue contraction at the four points based on the type of malocclusion. Similarly, there is no discernible gender difference in lip and tongue contractions at any of the four points in Table 2 . This table also demonstrates that there is no discernible age-related variation in lip and tongue muscle contraction. EMG images of tongue and lip muscle strength are shown in Figs. 5 and 6 .

**Fig. 7:**
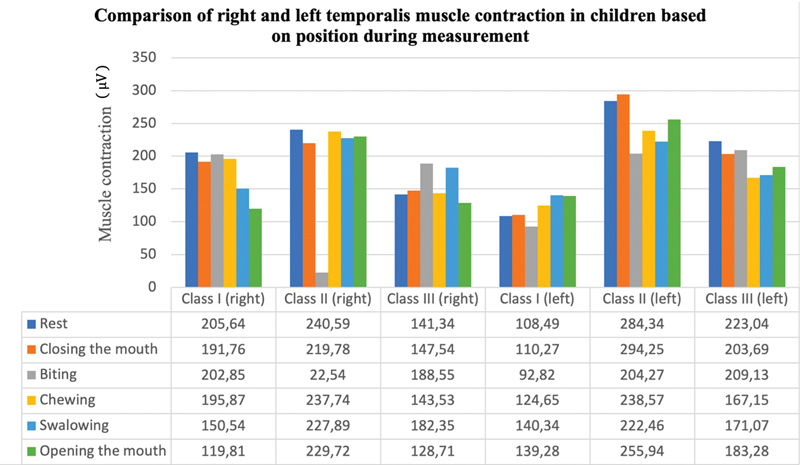
Comparison of right and left temporalis muscle contraction in children during various conditions and malocclusion class I, II, and III.

**Fig. 8:**
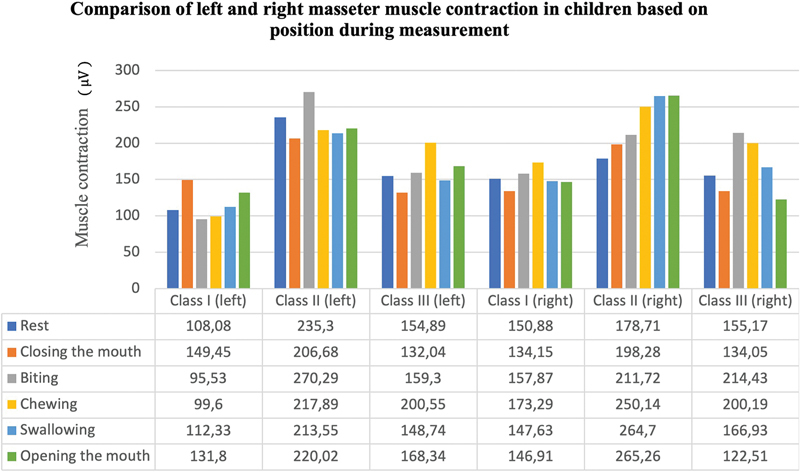
Comparison of left and right masseter muscle contraction in children during various conditions and malocclusion class I, II, and III.

Table 3 displays the findings of measurements of the contraction of the temporalis and masseter muscles on the right and left sides based on malocclusion in five movements: biting, chewing, swallowing, opening the mouth, and closing the mouth. The left temporalis muscle contracted differently when the subject was at rest, closed their mouth, and bit them. The left masseter muscle contraction during resting, biting, chewing, and swallowing was also found to differ significantly. The difference in contraction of the right and left temporalis and masseter muscles is shown in Figs. 7 and 8 .

## Discussion

Surface EMG electrodes utilize a noninvasive approach for EMG signal measurement and detection. It is theorized that between the skin of the body and the detecting surface, there is the establishment of a chemical equilibrium that occurs through electrolytic conduction. 1 31 32 33 34 EMG analysis provides important information about the condition of muscles, both in dynamic and static contractions. 29 35 36 Several authors have analyzed EMG characteristics under dynamic conditions in subjects with and without malocclusion. EMG is considered the most objective and reliable diagnostic tool for assessing changes in the electrical activity of masticatory muscles. This illustrates how malocclusion and muscular activity are related. 30 34 37

Different malocclusions (class I, II, and III) can alter muscle activity, which can be a predisposing factor for more severe malocclusions. 30 To determine the value of muscle activity, data analysis was performed by examining the relationship between the frequency and intensity of the recorded electrical activity. The units used for capturing electrical signals are microvolts (µV). 38

This study examines the muscle activity of the lips, tongue, temporalis and masseter masticatory muscles at rest, closing the mouth, biting, chewing, swallowing, biting, and opening the mouth using EMG equipment in class I, II, and III malocclusions in children aged 6 to 17 years to detect malocclusion. The data analysis results ( Table 1 ) indicate that there are no differences in the contraction of the tongue and lip muscles to detect class I, II, and III malocclusions. These findings are in line with research that has not discovered any differences in lip and tongue pressure or resistance among individuals with different malocclusion classifications. 39 40 41 42 43

Class II division II malocclusion subjects exhibited high lip strength, whereas Class II division 1 subjects exhibited lower lip strength, according to research by Lambrechts et al. 44 Incompetent lips are the result of a protruding maxilla and retrusive mandible in class II division 1 malocclusion. 45 The hypotonic state of the lip muscles and their innate elasticity are indicated by the lower EMG activity at rest in children with incompetent lips than in those with competent lips. 46 Class II malocclusion was not categorized in this study according to its division, making it impossible to distinguish. This study only looked at pediatric age, whereas the study by Lambrechts et al looked at both adult and pediatric patients. 44 Martins et al claim that during craniofacial growth and development, lip strength and endurance in subjects with malocclusion adapt to fulfill their function as the subject gets closer to adulthood. 41

Based on age and gender, there is no difference in the tongue and lip muscle contractions, according to the data analysis results ( Table 2 ). This is consistent with Yu and Gao's research, which found no gender-related differences in tongue muscle pressure during the resting phase. But when it came to the maximum tongue pressure used when chewing, women's results differed significantly from men's. 47 Additionally, a study by Lee et al revealed no significant difference in lip muscle compressive power and gender. However, there was a noticeable difference in the tongue muscles, with men having a higher compressive power. 48

Men and women have different lip contraction strengths, men have stronger lip strength than women. 44 This is because men and women have different amounts of muscle mass. 48 The performances of oral motor function and tongue pressure have an impact on the strength of the lip in both men and women. 49 Furthermore, environmental and general factors also impact lip function. For instance, a weaker lip is linked to an overrepresentation of the C allele for markers in the ACTN3 gene. 50 51

Lip strength does not differ by age in a healthy population. A study by Clark and Solomon compared lip compression strength in three age groups: young (18–29 years), mature (30–59 years), and old (60–89 years). There was no difference in lip strength among the three groups. The maintenance of lip strength in older age is due to the activity of the lip muscles during daily chewing and swallowing. 52 53 54 The increase in skeletal muscle strength with training is accounted for by changes in muscle structure, including muscle mass, cross-sectional area and fiber type composition, as well as changes in neural adaptation. 55

From the results of this study, it was found that there was a significant difference in the left masseter muscle and left temporalis muscle. According to research by Ramsundar et al, 56 there is no significant difference between the right and left temporalis and right and left masseter muscles in subjects with an overjet of more than 4 mm. The significant difference in the left masseter muscle and left temporalis muscle could be due to tooth decay on one side, for example, hypomineralization of the incisive molar. Children with molar incisor hypomineralization (MIH) have impaired crushing and chewing of food, as evidenced by muscle hyperactivity. Instabilities in masticatory biomechanics have induced greater muscle effort, and required greater muscle fiber recruitment, when compared with children without MIH, leading to muscle hyperactivity and decreased masticatory efficiency. 57

The significant difference on one side is also due to the crossbite on one side of the jaw, i.e., decreased masseter activity on the crossbite side and increased masseter activation on the contralateral side, i.e., lack of muscle coordination between sides, among the chewing patterns of the crossbite side. As a result, the masseter muscle on the unaffected side is more loaded than the masseter muscle on the crossbite side. 58 59 The impact of unilateral electrical activity on the temporal muscle is more closely related to the contralateral side, while on the masseter muscle the effect is bilateral. 60 Statistical analysis showed that the greater the force applied, the higher the electrical activity in the muscle. The significant difference on the left side may be due to overbite on the left side, affecting the left masseter muscle. 60

This study also showed that left temporalis contraction in class II malocclusion had a high value compared with class I and III when closing position. The class II malocclusion group exhibited higher average clinical results of temporalis muscle activity than the class I malocclusion group. Our findings support the findings of Petrović et al's research, which indicated that masticatory muscle activity in class II dental malocclusion was higher than in class I dental malocclusion with normal occlusion due to an excess of masseter and temporal muscle activity. 61 62

According to other research, compensatory muscle activity is frequently present in class II division II anomalies, particularly in the posterior fibers of the masseter and temporalis muscles. Eighty percent of patients with Angle class II division II anomalies had increased masseter muscle electromyographic activity. This correlates with the masticatory pattern, which is frequently observed because of the predominance of vertical mandibular movement, and is caused by increased activity of the mandibular elevator muscle. 63 The maximum bite force in the intercuspal position exhibited a highly significant positive correlation in the younger age group, suggesting that bite force increases with age. People who bite their incisors more forcefully also bite their molars more forcefully. 64

## Limitations of the Study

The main limitation of this study is the variable. In this study, the variables of permanent and primary teeth were not included.

## Conclusion

EMG can be considered as a tool to detect class I, II, and III malocclusion through muscle contraction, where biting and chewing positions have satisfactory EMG examination results to detect malocclusion. The age and gender of the child can affect the results of EMG examination in certain conditions.
